# Implementation of Low-Level Laser Therapy in Dentistry: A Review

**DOI:** 10.7759/cureus.28799

**Published:** 2022-09-05

**Authors:** Aishwarya Rathod, Priyanka Jaiswal, Pavan Bajaj, Bhairavi Kale, Deepika Masurkar

**Affiliations:** 1 Periodontics, Sharad Pawar Dental College, Datta Meghe Institute of Medical Sciences, Wardha, IND

**Keywords:** low-level laser, low-level laser therapy, phototherapy, emission, lasers, biostimulation

## Abstract

A type of light therapy known as low-level laser therapy (LLLT) uses only one wavelength of light. Low-level lasers (LLL) do not have a warming effect on the tissues; instead, they have an effect called photobiostimulation. LLL do not evaporate the tissue. The use of LLL to manage a range of illnesses is known as LLLT. Helium-neon lasers are an illustration of an LLLT product. Gallium arsenide, the infrared semiconductor made of gallium aluminum arsenide, is also an example. The performance powers range from 50 to 500 mW with electromagnetic spectrum wavelengths in the red and near-infrared region spanning from 630 to 980 nm and pulsed or continuous-wave emission. In periodontics, LLLT has gained prominence for several applications, including wound healing and pain relief after non-surgical and surgical procedures.

## Introduction and background

The term "laser" refers to the amplification of light by stimulated and excited emission or the amplification of excited light distribution. Photons are excited by atoms, which leads to the release of more photons and, eventually, the generation of a strip of homologous, mono-color, and parallel light known as a "laser." Theodore Maiman invented the laser on May 16, 1960, by the application of the synthetic ruby crystal. The earliest application of lasers in dentistry was for oral soft tissue surgery. The laser performed the function of a scalpel by ablating or cutting tissue with its energy. For it to function, a synthetic substance inside a light chamber is stimulated to produce bright light. To release more photons, photon radiation is excited by atoms. The process ends with the creation of a laser, which is a strip of homologous, monochromatic, and parallel light [1].

The present attention on the biological and medical consequences of very low levels of laser light is a result of Mester's work in Hungary in the early 1970s [2]. The majority of the initial research was done in Eastern Europe, and it was not initially given much credence in the West. However, times have changed, and a considerable amount of research has appeared in recognized American and European periodicals. Low-level laser therapy (LLLT) is currently covered in the majority of conferences on clinical laser applications due to its high level of interest [3]. The use of LLLT in dentistry is not new, and LLLT procedures have been used extensively in Europe and Japan for more than 10 years. The terms "mid-laser" and "soft-laser" are two of the more well-known names for the therapy technique. LLLT is defined as a procedure with a dose rate that does not result in either a macroscopically visible change in tissue structure or an immediately noticeable increase in tissue temperature [4].

An increase in temperature may be noticeable in actual practice, but it is frequently considered significant enough to be neglected. This hypothesis can be verified in comparison studies where a similar temperature increase is induced by means other than exposure to visible light [5]. The second reason why many people are reluctant to accept that LLLT can genuinely have clinical effects is the lack of a logical justification for the mechanism. Karu's studies [3,6] suggest that biological molecules have absorption bands at optical wavelengths and that exposure to light at different wavelengths can influence the rate at which particular biochemical processes take place. Recent research has demonstrated that laser light can have an impact on the tissue levels of copper and zinc. These two metals are necessary for the activity of enzymes. There have been proposed mechanisms for how polarized light affects the molecules in cell membranes [7,8].

LLLT is now a widely used adjuvant medical treatment for wound healing. The primary principle of LLLT is based on the bio-stimulating outcome [9]. It is based on the idea that exposure to a specific wavelength can alter cellular behavior [10]. As a result, both cell metabolism and proliferation increase. The excellent physical properties of LLLT, such as its bactericidal effect, cell stimulation, ablation, and fibroblast proliferation [11], hemostasis-enhancing chemotactic activity of leukocytes [12], and proliferation, differentiation, and calcification of osteoblastic cells are thought to make it an effective treatment for periodontal diseases [13]. Harrel and Rees [14] created a minimally invasive technique to produce minimal flap reflection, minimal incisions, and sensitive treatment of the periodontal tissues in intrabony disorders. In 2007, Trombelli introduced a single flap approach for treating intrabony defects [15]. By elevating the restrained mucoperiosteal flap on the lingual or buccal side for surgical access, the surrounding gingival tissues are protected in this procedure. As a result, the gingival aesthetics are preserved, the gingival vascular supply is restored, the detached papilla is stabilized, the primary intention of the wound is adequately closed, and healing occurs. Other dental specialties, such as the treatment of temporomandibular joint disorders, can benefit from the use of LLLT [16,17].

## Review

Low-level laser therapy

LLLT, also referred to as "soft laser therapy," has been applied in medicine for more than three decades. Low-level lasers (LLL) emit short-wavelength red or infrared light with a low water absorption power that can reach a depth of 3 mm to 15 mm in both soft and hard tissues [1]. Laser phototherapy (LPT), biostimulation therapy (BT), and low-intensity laser treatment (LILT) are other names for LLLT. LLLT stimulates the body's cells by delivering direct biostimulation light energy to them [18]. The molecules and atoms in cells are stimulated by the absorption of laser radiation. The temperature of the tissues is not significantly and quickly raised when low-intensity laser radiation is applied to them. LLLT has been demonstrated to aid in the management of pain, wound healing, and nerve injury. LLLT is also known as biostimulation or biomodulation [19]. The biostimulatory activity of laser irradiation results in physiological, metabolic, and functional changes in living microorganisms. The first biostimulation laser to be sold commercially was a 1-mW helium-neon (HeNe) laser. The most used LLLT laser in dentistry is the gallium-aluminum-arsenide laser (780 and 830 nm). The molecular signal of the cell changes as a result of photon absorption, which causes a change in the photoacceptor molecular structure. Modifications in the photoacceptor function are the main reactions. Cellular signals and functioning can change as a result of secondary reactions [20].

Mechanism of action of LLL

The biostimulatory effect of laser irradiation results in physiological, metabolic, and functional changes in living microorganisms. A 1-mW HeNe laser was the first commercially available biostimulation laser. The most used LLLT laser in dentistry is the gallium-aluminum-arsenide laser (780 and 830 nm). Photon absorption causes a shift in the molecular configuration of the photoacceptor, accompanied by an associated alteration in the molecular signal of the cell. The alterations in photoacceptor function are the primary reactions and subsequent alterations in cellular signaling, and cellular functions are secondary reactions. According to some reports, laser-enhanced biostimulation promotes intracellular metabolic changes that speed up matrix production, fibroblast migration, and cell division. The amount of light energy that is absorbed impacts the laser's ability to stimulate biological processes. The depth of energy penetration is biased by several factors, including wavelength, optical and temperature features, strength, energy values, exposure time, waveform optical properties of tissue absorption, and scattering coefficient [21,22].

LLL's impact mechanism on inflammation

LLL can minimize inflammation and the appearance of MMP8 (matrix metallopeptidase 8) after scaling. Additionally, it can inhibit the production of prostaglandins and increase plasminogen activity. Studies have specified that LLL can reduce interleukin 1 beta (IL-1β), although the result depends on the length of the radiation. Interferons are simultaneously suppressed while the production of platelet-derived growth factor (PDGF) and transforming growth factor beta (TGF-β) is stimulated. These two changes would cause LLL to have an anti-inflammatory impact, which would explain how it promotes wound healing. Lasers with a 670-nm wavelength that are combined with conventional periodontal treatments lead to better treatment outcomes and shorter treatment times [9].

LLL's impact mechanism on repair

LLLT causes vasodilation, local blood circulation, and relaxation of the soft vascular muscles. Vascular dilation also results in better blood flow and immune cell migration to the tissues, both of which accelerate tissue repair. On the other hand, LLL can open up blood arteries by affecting mast cells. The present evidence is very good for the capacity of lasers with the wavelengths of 820 nm, 940 nm, and 660 nm to induce mast cell degranulation and the consequent release of pro-inflammatory tumor necrosis factor (TNF) to accelerate leucocyte dispersal in the tissue. On the other hand, the basic membrane will be altered by the protease that mast cells create, making it easier for leucocytes to enter the tissue. When exposed to LLL, lymphocytes get activated and multiply more quickly. LLL exposure causes fibroblasts to reproduce and mature more frequently, change from fibroblasts to myofibroblasts, produce less E2 prostaglandin, and produce more basic fibroblast growth factors (bFGF). It is important to remember that although LLL may have these effects on the epidermis, buccal mucosa, and gingival mucosa, excessive dosages of the substance prevent fibroblast proliferation and growth factor release [1,23].

LLL's impact on pain

LLL has been recommended as a pain-regulator protocol that has more advantages than oral pain relievers and anti-inflammatory non-steroidal tablets because the anti-inflammatory function of this type of laser overlaps with its ability to enhance wound repair. Though the precise mechanism is unknown, numerous studies have linked the physiological changes caused by light's interaction with different cells to cause analgesic effects. Amplification of adenosine triphosphate (ATP) synthesis, enhancement of the rejuvenation system, and firmness of the lipid bilayer membrane and its proteins are a few of the proposed mechanisms. LLLT can reduce inflammatory pain by moderating the inflammatory process in a dose-dependent manner. The best results come from recommending LLL for acute pain within the first 72 hours after surgery [24].

Application of LLLT in dentistry

Soft Tissue Application

In cases of aphthous ulcers, according to several reports, patients who received LLLT therapy showed substantially less pain and functional complications. Furthermore, they claimed that as compared to traditional drug therapy, they experienced faster healing. On epithelial cells, no adverse effects have been identified. In herpes simplex infections, research has shown that LLLT has a positive effect on the healing of herpes simplex infections. Patients were given LLLT therapy every day for two weeks in one of the trials. Patients who underwent laser therapy had an average period of 37.5 weeks without herpes lesions, while patients who did not receive laser therapy had an average interval of three weeks. In oral lichen planus, studies have shown that using LLLT to treat oral lichen planus reduces discomfort and reduces the size of the lesions significantly. The treatment had no side effects and was equally as effective in treating lichen planus as topically applied corticosteroids. The use of an infrared laser significantly increases salivary flow in xerostomia patients. A 904 nm laser was used in the parotid and submandibular glands to activate the cells, and the results showed that it was successful in lowering xerostomia. Mucositis is a common complication in patients who have received chemotherapy or radiotherapy for cancer treatment. Oral mucositis and the daily mucositis index scores were considerably reduced when LLLT was administered daily inside the oral cavity. Additionally, it led to fewer xerostomia side effects, reduced pain levels, and improved swallowing abilities as compared to non-laser therapy patients. Paresthesia is one of the risks associated with surgical treatment in dentistry, which is most often seen through the extraction of third molars. Based on their findings, researchers concluded that using a gallium-aluminum-arsenide (GaAlAs) diode laser with 820-830 nm wavelength for 90 seconds in 20 applications during each treatment improved mechanoreceptive perception in the damaged alveolar nerve significantly [25]. One of the most prevalent conditions affecting the tooth's attachment system is periodontitis. It is the main reason why elderly people lose their teeth. Obradovic et al. found that when periodontal disease patients were treated with LLLT (670 nm) in addition to traditional periodontal therapy, healing and collagenization improved [26]. Lower-level soft tissue lasers have been shown to have analgesic benefits in the literature. The impact is thought to be due to endorphin production being stimulated or interference with the mediation of pain impulses. Lower-intensity soft tissue lasers have been shown to have analgesic benefits in the literature. The impact has been described in terms of interfering with the transmission of pain impulses and/or stimulating the synthesis of endorphins. The lymph vessel's permeability is reduced by LLLT, which can also stimulate the lymph vessel's collaterals. By increasing phagocytosis and expanding the number and size of lymph vessels, edema may be reduced [27].

Hard Tissue Application

Dentinal hypersensitivity (DH) is one of the most typical reasons for dental discomfort. It reduces hypersensitivity with LLLT and uses changes in neural transmission networks caused by lasers in the dental pulp. In the majority of DH investigations, GaAlAs laser therapy has been used to desensitize cervical dentine that is overly sensitive, with a 90% success rate. Patients with myofascial pain dysfunction syndrome saw a significant change in mandibular movement after receiving LLLT care (820 nm). Active and passive mouth opening, as well as right and left lateral movements, have all temporomandibular disorders been recorded to improve after LLLT. LLLT has been shown in trials to help manage the pain associated with orthodontic post-adjustment. Studies have revealed that LLLT reduces the release of highly pro-inflammatory chemicals [28]. It is also used for bone remodeling. The stimulation of mesenchymal cells or the direct stimulation of osteoblasts results in low-level laser bioregulation of bone formation. It is conceivable that biostimulation results from an elevated release of the fibroblast growth factor that is contained in bone tissue. It affects differentiated cells, promoting both cell proliferation and the release of matrix components [29]. Increased use of acidic foods and gastroesophageal reflux have both contributed to an increase in the prevalence of erosion. LLLT may be an alternative strategy because its application may cause changes to the organic matrix content of enamel, which could boost the material's resistance to demineralization [30]. LLLT has a stimulatory effect on root development. Caries and dental trauma can stop the growth of roots by causing pulp necrosis. In addition to enhancing the synthesis of collagenous and non-collagenous proteins from the extracellular matrix (ECM), LLLT speeds up tissue recovery by forming a fibrous matrix and dentin bridge. During the various stages of dentinogenesis, these proteins can act in differentiation, migration, and proliferation, in addition to playing a significant part in the production of mineralized tissues [31]. The success of an implant is mostly determined by osseointegration. Numerous applications of LLL in peri-implant tissue issues increase the number of viable osteoclasts and metabolic bone activity by promoting local blood flow, increasing the area of bone that contacts the implant, and accelerating bone maturation. It changes the bone-implant contact surface area to speed up osseointegration [32].

Advantages

Laser therapy has been demonstrated to reduce pain and inflammation. One of the most prevalent musculoskeletal ailments is temporomandibular joint disorder (TMJD). Low-level light speeds up the body's normal healing process without burning the skin. Chronic and recent injuries can be successfully treated with LLLT. LLLT is a fantastic, healthy, natural, and effective alternative if you are sensitive to or allergic to harsh medications [33].

Disadvantages

The disadvantages are that, after the first therapy, patients frequently do not completely recover from their pain. Patients are often required to visit the doctor for care at least twice a week. Old injuries may worsen for a few days after procedures; however, most patients only have this discomfort for a short time [33].

LLLT application in periodontology has been explained in various studies with different cases as mentioned in Table 1. The procedures like root coverage, intraosseous defect, gingivectomy, implant, furcation involvement, and non-surgical periodontal therapy can be easily done with LLLT. Table 2 shows explanations of the uses of LLLT in the dentistry field. As dentistry is a wide field with various specialties, the uses of LLLT are in orthodontics, pedodontics, oral surgery, endodontics, and oral medicine.

**Table 1:** Role of LLLT in periodontics LLLT: low-level laser therapy; CAF: coronally advanced flap; MRTD: multiple-recession type defects; CAL: clinical attachment level; LLL: low-level laser; LILT: low-intensity laser therapy; PPD: periodontal probing depth; InGaAsP: indium gallium arsenide phosphide.

Periodontal surgery	Study	Conclusion	
Root coverage	For the treatment of multiple-recession type deformities, Ozturan et al. (2011) evaluated the effects of LILT concerning root coverage after CAF operation (MRTD) [34].	Notwithstanding the study's shortcomings, the authors found that LILT may enhance CAF prediction in several recessions.	
Intraosseous defect	Bhardwaj et al. studied LLLT in the treatment of intraosseous defects [35].	The combined LLLT and demineralized bone matrix of the bovine origin approach produced favorable outcomes in terms of CAL growth, a reduction in periodontal probing depth (PPD), low clinical recession, linear bone gain, and bone filling on radiographs.	
Gingivectomy	In this split-mouth controlled clinical experiment, Kohale et al. (2018) evaluated the impact of LLLT employing a diode laser (InGaAsP) on patients' responses and wound healing following scalpel gingivectomy [36].	The authors concluded that, within the constraints of the study, the findings suggested that LLLT might accelerate gingivectomy wound healing.	
Implant	The impact of 650 nm LLL irradiation on the decline in problems following advanced implant operations was examined by Pouremadi et al. in 2019 [37].	Adjuvant laser therapy could considerably enhance wound healing and lessen the intensity and length of pain and edema about the biological consequences of sophisticated implant operations and associated problems.	
Furcation involvement	All investigated the clinical and radiographic effects of LLLT on the recovery of human grade II furcation involvements treated with xenograft materials derived from cattle in a study published in 2015 [38].	The data pointing to possible therapeutic benefits of LLLT for periodontal tissue regeneration for furcation grade II lesions suggest that LLLT for furcation grade II lesions may promote new bone production and bone density.	
Non-surgical periodontal therapy	LLLT was examined by Gündoğar et al. (2016) as a supplement to the non-surgical treatment of chronic periodontitis [39].		The authors concluded that adding LLLT to non-surgical periodontal treatment improves clinical metrics.

**Table 2:** Role of LLLT in dentistry LLLT: low-level laser therapy; TMD: temporomandibular disorders; LPBM: laser photobiomodulation; OTM: orthodontic tooth movement.

LLLT in dentistry	Study	Conclusion
Orthodontics	LLLT was examined by Guram et al. (2018) about the length of orthodontic tooth movement (OTM) and pain perception [40].	LLLT can lengthen the fixed OTM timing and lessen discomfort felt during orthodontic treatment.
Pedodontics	Yavagal et al. (2022) assessed and contrasted the clinical and radiographic success rates of formocresol pulpotomy and laser photobiomodulation (LPBM) at nine months following intervention in human primary molars [41].	In comparison to formocresol pulpotomy, the radiographic success rate of LPBM pulpotomy was noticeably higher, demonstrating that LPBM is a potential pulpotomy approach for young patients.
Oral surgery	In a systematic review and meta-analysis, Domah et al. (2021) examined whether LLLT is superior to placebo in lowering postoperative morbidity in individuals having their mandibular third molars surgically removed [42].	In comparison to a placebo, LLLT considerably lowers edema following mandibular third molar extraction. There is no evidence that LLLT can lessen trismus and postoperative discomfort. LLLT has no negative side effects. Evidence is currently lacking to support the investment in LLLT vs. the net clinical benefit. To offer firm recommendations to doctors regarding its use on patients, randomized controlled studies with bigger sample sizes, standardized study designs, and outcome measures are needed.
Endodontics	Metin et al. (2018) investigated the potential advantages of LLLT on the recovery of soft and hard tissues following endodontic surgery [43].	According to this study, LLLT helped soft and hard tissues heal after endodontic surgery and improved patient discomfort and quality of life, especially in the early phases of recovery.
Oral medicine	A systematic review by Maia et al. (2012) examined the impact of LLLT on people with TMDs' pain thresholds [44].	The majority of studies indicated that LLLT appeared to be useful in easing TMD discomfort. Interpreting these data, however, requires caution due to the heterogeneity of the standardization for laser parameters. Therefore, more studies must be done to reach a consensus on the appropriate application procedure for pain alleviation in TMD patients.

Contraindication

LLLT is contraindicated in treating directly over the growing fetus in pregnant women. Patients with epilepsy who are photosensitive should be informed that low frequency pulsed visible light (30 Hz) may cause a seizure. Patients with pacemakers are not prescribed LLLT, and it should only be used cautiously. It should not be used in patients with chest pain [45].

## Conclusions

In many dental specialties, LLLT is a useful adjunctive treatment. On the hard and soft tissues of the oral cavity, it has a positive effect with fewer side effects. Another requirement is to assess the effectiveness of the LLLT examined in relation to the site of application, wavelength, treatment time, and dosage. An increasingly popular method of therapy in the medical industry is laser radiation. Therefore, it is crucial to analyze and provide evidence for the underlying physiological impacts and risks. Numerous physiological consequences have been observed; these effects need to be further researched, and the implications for therapeutic practice must also be taken into account. LLLT's possible risks are likewise unknown. Safety for both patients and doctors is crucial, so this matter needs to be carefully considered. In conjunction with non-surgical periodontal therapy, photobiomodulation, a process mediated by low-level lasers, promotes additional benefits in the near term, speeds up the healing of bone and gingival tissue, and lessens the side effects of periodontal surgery. In the initial re-evaluation phases, the impact of photodynamic therapy on microbes is important. LLLT can provide patients with a wide range of therapeutic advantages, including faster wound healing and pain alleviation. There is still much to learn about the mechanisms, identifying the therapy window, and using these biological phenomena correctly to achieve the treatment objectives.
